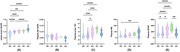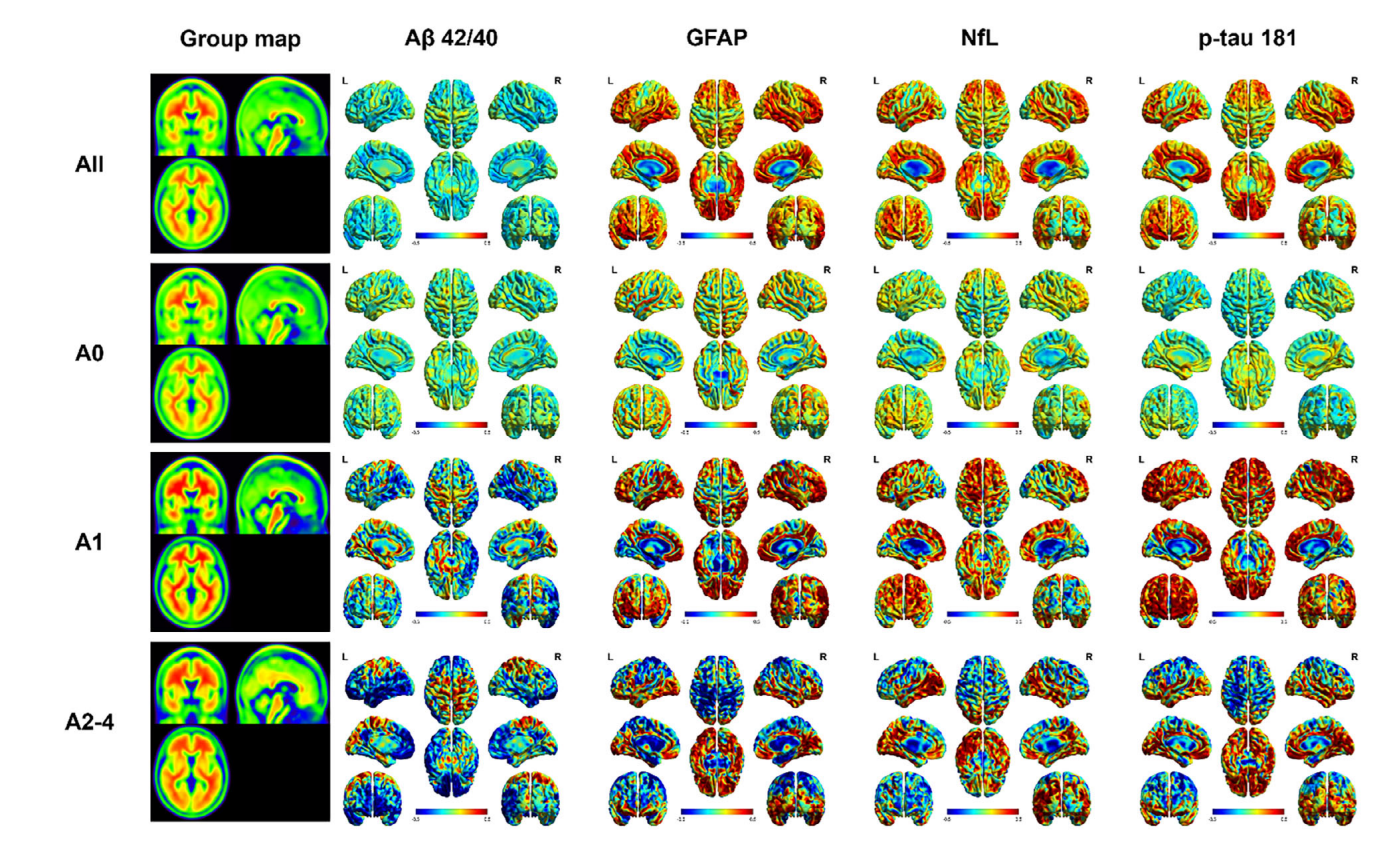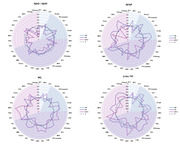# Effect of early Alzheimer's disease plasma markers on Aβ deposition: Evidence from SILCODE

**DOI:** 10.1002/alz.086351

**Published:** 2025-01-09

**Authors:** Xianfeng Yu, Xia Zhou, Rong Shi, Jiehui Jiang, Ying Han

**Affiliations:** ^1^ XuanWu Hospital of Capital Medical University, Beijing China; ^2^ the First Affiliated Hospital of Anhui Medical University, Hefei, Anhui China; ^3^ School of Communication and Information Engineering, Shanghai University, Shanghai, 200444, China, Shanghai, Shanghai China; ^4^ Institute for Advanced Communication and Data Science, Shanghai University, Shanghai, Shanghai China; ^5^ Department of Neurology, Xuanwu Hospital of Capital Medical University, Beijing, Beijing China

## Abstract

**Background:**

Previous studies have found that AD‐related plasma markers are associated with Aβ deposition, but the specific effects remain unclear.

**Method:**

Data were obtained from the Longitudinal Study of Cognitive Decline in China (SILCODE). Comprehensive neuropsychological assessments, MRI, plasma samples, and amyloid positron‐emission tomography (Aβ‐PET) data were collected. Through the SUVR value calculation and Aβ stage classification, as well as the correlation analysis between plasma biomarkers and voxel /ROI SUVR values and clinical scale information, respectively, mediation analysis was used to study the possible pathways.

**Result:**

This study involved 148 participants from Xuanwu Hospital in Beijing, including 66 cognitively normal (CN), 59 subjective cognitive decline (SCD), 12 MCI and 11 AD patients in this dataset. The proportion of CN and SCD in stage A0‐1 was the highest, while A2‐4 MCI and AD increased. Analysis of variance showed that except Aβ42/Aβ40, the other three (p‐tau181, GFAP and NfL) plasma biomarkers had differences between groups, and GFAP showed significant differences in each stage. Plasma p‐tau181 was significantly lower in stage A0 and A1 than in stage A2 and A3. Plasma GFAP at stage A0 was significantly lower than that at stages A1, A2, and A3, and at stage 1, it was significantly lower than that at stages A2 and A3. Plasma GFAP at stage A3 was significantly higher than that at stage A4. There were two pathways that showed significant fully mediated effects: in the PET SUVR‐ plasma p‐tau181‐MMSE score pathway, all four meta‐ROI SUVR showed fully mediated effects, while in the PET SUVR‐ plasma GFAP‐MMSE score pathway, with only 2 meta‐ROI SUVR values, stage A2 and stage A4 showed mediation effects.

**Conclusion:**

The present study demonstrated the role of plasma in the early stage of AD, especially SCD, from the clinical diagnosis and Aβ stage dimensions. Studies have found that P‐tau181 has a good hint role throughout the course of AD, while GFAP may have a ceiling effect in the middle stage of AD and a significant hint role in the early and late stages of AD.